# Case of Organic Disease of the Heart, with Dissection

**Published:** 1814-10

**Authors:** John Gorham


*For the Medical and Physical Journal.*

### *Case of Organic Disease of the Heart, with Dissection; by* JOHN GORHAM, M.D.

THE subject of this communication was a negro of an athletic form, and apparently about forty years of age. He came under my care in March, 1813. He entered the alms-house in December 18.2, for a syphilitic complaint, and had been attended there by Dr. Jackson, for cough and difficulty of respiration, increased by exercise and by lying in in a horizontal position; these were accompanied with a tense, frequent, and occasionally an irregular pulse, swelling of the abdomen and feet, pains about the neck and shoulders, and paucity of urine. No pulse could be perceived in the right arm. He had been treated with quicksilver, expectorants, and diuretics.

The symptoms, when I took charge of him, were more developed. The cough was hard and incessant, accompanied with expectoration of frothy mucus, constant dyspnœa, violent palpitations of the heart, frightful dreams, from which he awoke in extreme terror and with a sense of suffocation; pulse irregular and often intermittent; swellings of the lower extremities and abdomen, and, a few days previous to his death, of the face and upper extremities; distressed and harassed countenance; urine high-coloured, and very small in quantity; and total want of appetite. He uniformly complained of pain and great distress at the stomach. On the 8th of April, his pulse, though irregular, was faint. I prescribed some medicine for him, but in five minutes he was dead.

*Examination of the body twenty-four hours after death.*— The left side of the thorax, when struck, resounded; the sound returned from the right side was like that produced by striking a solid substance. The muscles were of a dark colour. On opening into the thorax, the right side was found filled with transparent yellowish serous effusion. On the anterior and superior portions of the lungs were signs of slight inflammation. There was no adhesion, and the organ was apparently healthy. When divided by the knife, a considerable quantity of frothy blood-coloured fluid issued from the cut surfaces.

The left cavity of the lungs contained six or eight ounces of yellowish fluid. The lower portion of the lung was sound; the upper portion was white and crispy, and adhered by two membranous bands to the mediastinum; when cut into, it presented the same white appearance, and was much less firm than the other portions.

The substance of the pericardium was not thicker than usual. On its upper surface, there had been an effusion of lymph, which had formed a tough and elastic but thin membrane, about one inch in length, and half an inch in breadth. When opened, the cavity was found to contain about six ounces of a pellucid serous fluid. The heart, thus brought into view, appeared nearly of twice the natural size, and very turgid. The coronary veins were exceedingly distended, distinct, and prominent. On the superior surface there were appearances of effusion of lymph, particularly around some of the smaller veins, the coats of some of which were white, and much thickened. The right auricle and ventricle were distended with coagulated blood; the semi-lunar valves of the pulmonary artery were sound. The left ventricle was filled with blood, for the most part fluid; its walls were thick and firm. The semi-lunar valves of the aorta were completely ossified, and the aorta itself was thickened, and its coats filled with osseous matter from its origin in the heart to beyond its arch. The mitral valves and the coronary arteries were sound.

Abdomen. The colour of the liver was lighter than usual. On the convex surface, near the suspensory ligament, there was a small effusion of lymph, and a greenish-coloured tumour, about the size of a bean, filled with a fluid resembling in every respect bile. The gall-bladder was of the usual size, and its duct pervious. The texture of this organ was natural, but, when divided, a blood-coloured fluid oozed out in considerable quantities.

The stomach was large, much thicker than usual, and its internal coat highly inflamed, in some parts presenting the appearance of a diffused redness, deep coloured, in others of innumerable minute specks of a chocolate brown; and the whole surface was covered with a thick tenacious whitish matter, resembling an intimate mixture of pus and mucus. With the exception of this substance, this organ was empty. The small intestines discovered signs of slight inflammation. The mesenteric glands were enlarged; the spleen and pancreas sound. The cavity of the abdomen contained two or three pints of clear yellowish fluid.

Cranium. The vessels of the membranes and brain were not unusually distended; and the only circumstance observed, which requires to be mentioned, was the effusion of serous fluid in considerable quantity into the ventricles.

The appearance of the stomach satisfactorily accounted for the constant pain and anxiety experienced by the patient in the epigastric region.—*New-England Journal.*

